# Transactions of Texas Medical Association, Eighteenth Session

**Published:** 1887-05

**Authors:** 


TRANSACTIONS OF THE TEXAS STATE MEDICAL ASSOCIATION, EIGHTEENTH ANNUAL SESSION, *April, 1886.*

This report of 691 pages gives a complete account of the proceedings of the State Society. The session was of four days' duration, and the reports af committees do not constitute the bulk of the matter presented. Much of the matter could be improved by condensation. The prize essay of the year, "Reflections on Physical and Mental Culture," is included in the transactions.

